# From Toxin to Treatment: A Narrative Review on the Use of Botulinum Toxin for Autonomic Dysfunction

**DOI:** 10.3390/toxins16020096

**Published:** 2024-02-10

**Authors:** Lucas Rempel, Raza N. Malik, Claire Shackleton, Martín Calderón-Juárez, Rahul Sachdeva, Andrei V. Krassioukov

**Affiliations:** 1 International Collaboration on Repair Discoveries, Faculty of Medicine, The University of British Columbia, Vancouver, BC V5Z 1M9, Canada; lucasrem@student.ubc.ca (L.R.); razamalik@icord.org (R.N.M.); shackleton@icord.org (C.S.); martincj@icord.org (M.C.-J.); rahulsac@mail.ubc.ca (R.S.); 2 Division of Physical Medicine and Rehabilitation, Department of Medicine, The University of British Columbia, Vancouver, BC V5Z 2G9, Canada; 3 GF Strong Rehabilitation Centre, Vancouver Coastal Health, Vancouver, BC V5Z 2G9, Canada

**Keywords:** botulinum toxin, autonomic dysfunction, bacterial toxin

## Abstract

Since its regulatory approval over a half-century ago, botulinum toxin has evolved from one of the most potent neurotoxins known to becoming routinely adopted in clinical practice. Botulinum toxin, a highly potent neurotoxin produced by Clostridium botulinum, can cause botulism illness, characterized by widespread muscle weakness due to inhibition of acetylcholine transmission at neuromuscular junctions. The observation of botulinum toxin’s anticholinergic properties led to the investigation of its potential benefits for conditions with an underlying etiology of cholinergic transmission, including autonomic nervous system dysfunction. These conditions range from disorders of the integument to gastrointestinal and urinary systems. Several formulations of botulinum toxin have been developed and tested over time, significantly increasing the availability of this treatment for appropriate clinical use. Despite the accelerated and expanded use of botulinum toxin, there lacks an updated comprehensive review on its therapeutic use, particularly to treat autonomic dysfunction. This narrative review provides an overview of the effect of botulinum toxin in the treatment of autonomic dysfunction and summarizes the different formulations and dosages most widely studied, while highlighting reported outcomes and the occurrence of any adverse events.

## 1. Introduction

Botulinum toxin (BoNT) injections are among the most commonly performed procedures in the world [1]. The rapid development of BoNT can partially be attributed to its publicized role in cosmetics, but its clinical application for various motor and autonomic dysfunctions have made it an appealing treatment option since the 1970s. The first classical accounts of BoNT were reported in 1817 when Justinus Kerner discovered sausage-borne BoNT and Emile Pierre van Ermengem described a BoNT outbreak in a Belgian village in 1897 [2,3]. It was not until the 1970s that BoNT was used for clinical use by Dr. Alan Scott for the treatment of strabismus [4]. In 1989, botulinum-A toxin (BoNT-A) was approved by the United States Food and Drug Administration (FDA) for treating strabismus, blepharospasm, and facial nerve disorders, and in 2000, the FDA-approved botulinum-B toxin (BoNT-B) for cervical dystonia [5]. The initial approved uses for BoNT were to target somatic cholinergic transmission at the skeletal neuromuscular junction. By the turn of the 21st century, new applications of BoNT were emerging, focused on treating autonomic dysfunctions [6]. The wide range of medical applications can be attributed to the multiple formulations of BoNT, each designed for specific clinical indications. 

## 2. Mechanisms and Formulations of Botulinum Toxin

**Figure 1:**
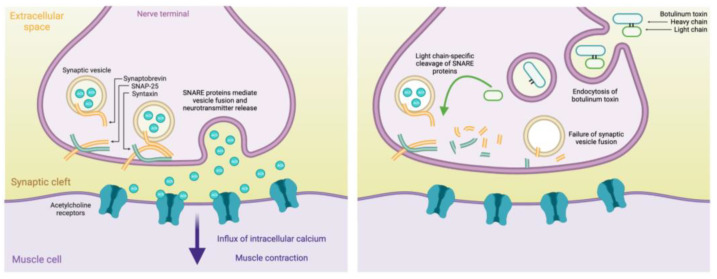
The effect of botulinum toxin on cholinergic signaling at the synaptic cleft. Comparison of signaling at the synaptic cleft between cholinergic nerve terminal and muscle. Left: in the absence of BoNT, synaptic vesicles containing acetylcholine fuse with the presynaptic nerve terminal membrane mediated by SNARE proteins (synaptobrevin, SNAP-25, syntaxin). Acetylcholine is released into the synaptic cleft where it binds to post-synaptic acetylcholine receptors, leading to muscle contraction. Right: when BoNT is present, it is endocytosed into the cholinergic nerve terminal. Botulinum toxin light-chain cleaves SNARE proteins, resulting in the failure of synaptic vesicle fusion and acetylcholine release. Created with Biorender.com.

Endogenous BoNT is produced by several bacteria belonging to the Clostridium genus, the most well-known being Clostridium botulinum [7]. Together, they produce seven serotypes of BoNT (A–G). BoNT consists of a 100 kDa heavy- and 50 kDa light-chain protein joined by a disulphide linkage [8]. BoNT inhibits the presynaptic release of acetylcholine (ACh) at the neuromuscular junction, through light-chain mediated cleavage of SNARE proteins that are necessary for the release of ACh-containing vesicles [9,10,11,12,13]. Figure 1 illustrates the mechanism of action of BoNT.

Among the serotypes of BoNT, types A and B have the longest duration of action in vivo, ranging from weeks to months, making them ideal for clinical application [10]. Widely studied formulations of BoNT-A include onabotulinumtoxinA (Botox^®^, Allergan Inc., Irvine, CA, USA), abobotulinumtoxinA (Dysport^®^, Ipsen, Paris, France), and incobotulinumtoxinA (Xeomin^®^, Merz Pharmaceuticals GmbH, Frankfurt, Germany). BoNT-B formulations include rimabotulinumtoxinB, which exists as Myobloc^TM^ or NeuroBloc^TM^ (Solstice Neurosciences, Malvern, PA, USA) [10,14,15,16,17,18]. Table 1 provides a summary of these four formulations. Newer FDA-approved formulations with solely cosmetic indications include BoNT-A formulations prabotulinumtoxinA-xvfs (Jeuveau^TM^, Evolus Inc., Newport Beach, CA, USA) and daxibotulinumtoxinA-ianm (DAXXIFY^TM^, Revance Therapeutics, Nashville, TN, USA) [19,20].

## 3. Targeting Autonomic Dysfunction with Botulinum Toxin

The autonomic nervous system (ANS) facilitates involuntary neural control over a diverse array of physiological functions. The ANS is compartmentalized into three major divisions: the sympathetic nervous system, parasympathetic nervous system, and enteric nervous system [22,23,24]. The sympathetic and parasympathetic nervous systems consist of efferent components with distinct anatomical organizations, where each is responsible for communicating with effector organs within the viscera, vasculature, and skin [22,25]. These efferent pathways are comprised of a preganglionic neuron with a cell body in the central nervous system (CNS) and a postganglionic neuron with a cell body within autonomic ganglia located outside the CNS that innervates target tissues. Specifically, sympathetic preganglionic neurons arise from spinal cord levels T1-L2 and synapse with ganglionic neurons in prevertebral or paravertebral ganglia. Parasympathetic preganglionic neurons arise from the brainstem (within nuclei of the cranial nerves II, VII, IX, X) and spinal cord levels S2–S4, synapsing in ganglia close to their effector organ [23,24].

Transmission within both sympathetic and parasympathetic ganglia occurs via the transmission of acetylcholine (ACh) [24]. Postganglionic transmission by the majority of sympathetic postganglionic fibers occurs via the release of norepinephrine [22,24,26]. A small portion of postganglionic sympathetic neurons are cholinergic and release ACh at eccrine sweat glands and piloerector smooth muscles [27]. Sympathetic activation leads to responses including, but not limited to, increased blood pressure and heart rate, bronchodilation, decreased gastrointestinal motility and secretion, urinary retention, and sweating [23]. Postganglionic parasympathetic responses are determined by the transmission of ACh [28,29,30]. Parasympathetic activation leads to responses including, but not limited to, decreased heart rate, bronchoconstriction, increased gastrointestinal motility and secretion, and micturition [23]. 

**Figure 2:**
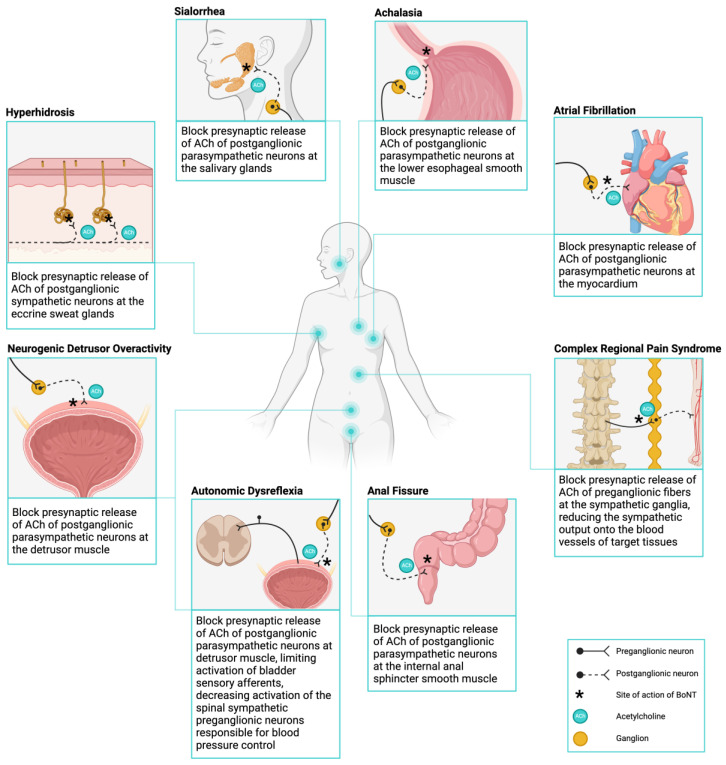
Targeting autonomic dysfunction with botulinum toxin. A summary of the sites where therapeutic botulinum toxin can aid in the management of a wide range of autonomic dysfunctions. The mechanism of action of botulinum toxin can be used to manage several conditions by inhibiting the presynaptic release of acetylcholine from sympathetic and parasympathetic neurons at their site of innervation with their effector organs. ACh, acetylcholine. Created with Biorender.com.

In general, autonomic dysfunction arises from perturbations to ANS signaling of idiopathic origin or associated with a known cause [31,32,33,34]. The usage of BoNT to treat autonomic dysfunctions lies in its ability to interfere with presynaptic cholinergic transmission within the sympathetic and parasympathetic postganglionic nerve fibers [10]. Figure 2 illustrates the potential sites of actions of BoNT to treat various autonomic dysfunctions.

## 4. The Use of Botulinum Toxin in Conditions with Autonomic Dysfunction

The following section discusses clinical evidence on the effects of different formulations and serotypes of BoNT on various autonomic dysfunctions. We implemented a narrative review protocol using prior published methodology [35,36,37]. A literature search was conducted using MEDLINE and PubMed electronic databases for studies published up until December 2022. Manual study searches were also performed using the reference list of articles during the search process. Included studies were clinical trials, case series, or case reports published in the English language before December 2022. Selected studies investigated the effects of botulinum toxin administered by injection to treat or manage autonomic dysfunction. Our search included the following keywords used in different combinations: “botulinum toxin”, “axillary hyperhidrosis”, “palmar hyperhidrosis”, “craniofacial hyperhidrosis”, “sialorrhea”, “achalasia”, “anal fissure”, “neurogenic detrusor overactivity”, “autonomic dysreflexia”, “atrial fibrillation”, “complex regional pain syndrome”. The selected literature is limited to the consensus of the authors. Table 2 summarizes the studies described.

### 4.1. Management of Hyperhidrosis

Hyperhidrosis is defined as excessive sweating as a result of increased sympathetic cholinergic activity on eccrine sweat glands, lasting six or more months [38]. It affects approximately 1–3% of the global population [38]. Hyperhidrosis can be classified based on distribution pattern into generalized and focal hyperhidrosis [38,39]. Generalized hyperhidrosis occurs over the entire body and can be primary (idiopathic) or secondary from a known cause, including infections, steroid use, menopause, hyperthyroidism, diabetes, obesity, and cancer [39,40]. Focal hyperhidrosis is localized to a specific areas of the body such as the axillae, face, palms, and soles. Similarly, focal hyperhidrosis can also be primary or secondary [39,40]. The major negative impact of hyperhidrosis involves the persistent softening of the skin from excess moisture, which can lead to skin maceration [40]. This increases the risk of cutaneous bacterial and fungal infections, particularly on the soles [40]. Other significant complications include psychosocial, as this condition can impact self-esteem, social interactions, relationships, and occupational choices [40]. 

BoNT is used in the treatment of hyperhidrosis by blocking sympathetic cholinergic input to eccrine sweat glands [38]. Currently, Botox^®^ is the only FDA-indicated treatment for severe primary axillary hyperhidrosis unmanageable with topical therapy [15].

#### 4.1.1. Management of Axillary Hyperhidrosis

Botox^®^ was demonstrated to be an effective treatment for axillary hyperhidrosis in two landmark clinical trials [41,42]. 50 U of Botox^®^ injected per axilla resulted in a ≥50% reduction in sweating from baseline [41]. The effect of a single treatment of Botox^®^ lasted up to 16 weeks, with repeated treatments resulting in a prolonged mean duration of effect of over 30 weeks [41,42]. Doses of 75 U of Botox^®^, demonstrated similar efficacy to 50 U [43]. Other formulations of BoNT, including 100–200 U Dysport^®^, 50–100 U Xeomin^®^, or 2000–4000 U of Myobloc^TM^ per axilla, have been demonstrated to all lead to sweat reduction ≥ 50% from baseline between 16–48 weeks [43,44,45,46,47].

#### 4.1.2. Management of Palmar Hyperhidrosis

Although there are no BoNT formulations that are FDA-approved to treat palmar hyperhidrosis, off-label injection of BoNT into the palmar surface has proved to be effective. Schnider and colleagues were the first to demonstrate this in an open-label clinical trial investigating palmar hyperhidrosis present from early childhood [48]. 120 U of Dysport^®^ significantly reduced palmar hyperhidrosis at follow-up tests 3-, 8-, and 13-weeks post-injection [48]. In addition, total doses of 25–150 U of Botox^®^, 120–284 U Dysport^®^, 25–150 U Xeomin^®^, or 5000 U Myobloc^TM^ have all been reported to decrease palmar sweating for ranges from 28 days to 24 weeks [48,49,50,51,52,53,54].

#### 4.1.3. Management of Craniofacial Hyperhidrosis

Craniofacial hyperhidrosis is defined as excessive sweating typically around the forehead, and less often around the upper lip, cheeks, and chin [55]. Currently, there are no FDA-approved BoNT formulations to treat craniofacial hyperhidrosis. Few studies have reported that craniofacial hyperhidrosis can be treated with off-label Botox^®^ in ranging doses from 10 U at the chin or upper lip, and up to 300 U for the scalp and forehead [55,56,57,58]. Secondary postmenopausal craniofacial hyperhidrosis, common in 10% of women 10 years after menopause, was also reported to be controlled with 100 U BoNT-A as well as a total of 2250 U Myobloc^TM^ [59,60,61]. 

Adverse systemic effects of BoNT for the treatment of hyperhidrosis are rare. Some studies reported no adverse events [45], whereas others reported local adverse events, including pain related to injection, temporary numbness, and weakness at the injection site [48,49,51,62].

### 4.2. Management of Sialorrhea

Sialorrhea is a condition characterized by excess saliva within the oral cavity pushing past the lip margin [63]. This can occur from an excessive production of saliva by the salivary glands or an inability to clear saliva from the oral cavity [63,64,65]. Salivation is primarily regulated by the parasympathetic branch of the ANS, involving the presynaptic release of acetylcholine at the glands [63,65]. The majority of saliva is produced by three pairs of major salivary glands: the parotid, submandibular, and sublingual glands [63,64,65]. Excess saliva production can be idiopathic, drug-induced, or condition-induced, whereas the inability to clear saliva from the oral cavity is commonly observed in neurological disorders such as amyotrophic lateral sclerosis (ALS), Parkinson’s disease (PD), cerebral palsy (CP), or stroke [63,64,66]. Sialorrhea can lead to physical complications, including an increased risk of perioral infection, dehydration, and choking [67]. Social stigmatization is a possibility, as those with sialorrhea may be unable to control their salivation and have to wear a bib towel to soak up excess saliva [67]. Currently, anticholinergic medications such as oral glycopyrrolate and sublingual ipratropium bromide are recommended to control sialorrhea in conditions like ALS [68,69]. 

BoNT injected at the salivary glands can mitigate presynaptic cholinergic signaling and limit saliva production in conditions like sialorrhea [64]. Currently, both Xeomin^®^ and Myobloc^TM^ are FDA-approved treatments for chronic sialorrhea [16,17]. The FDA-recommended total dose of Xeomin^®^ is 100 U (30 U per parotid gland and 20 U per submandibular gland) at most every 16 weeks [17]. The FDA-recommended total dose of Myobloc^TM^ is 1500–3500 U (500–1500 U per parotid gland and 250 U per submandibular gland) at most every 12 weeks [18].

The efficacy of BoNT as a treatment for sialorrhea was first demonstrated by Bhatia and colleagues in 1999 [70]. A 20 U injection of Dysport^®^ subcutaneously near the parotid gland reduced salivation to the point where patients no longer had to use a towel to collect saliva; this effect lasted for over six weeks. Dosage ranges of 50–200 U of Dysport^®^ per parotid gland have been studied in clinical trials, all resulting in similar improvements in reducing salivation. Doses of 200 U show the greatest effects, lasting for up to 24 weeks [71,72]. Greater immediate reductions in sialorrhea are also observed when a greater number of glands are injected in patients with sialorrhea from PD, stroke, or ALS [73]. In a comparative study of Botox^®^ and Xeomin^®^, Restivo and colleagues showed that while different injections of both BoNT-A toxins significantly reduced salivation to similar degrees, Botox^®^ was effective when four glands were injected versus three. Xeomin^®^ was effective when three glands were injected versus two [73]. Long-term BoNT injections of Xeomin^®^ are also effective at treating persistent chronic sialorrhea in PD without increasing the risk of adverse events over time [74,75]. Jost and colleagues administered either 75 U or 100 U of Xeomin^®^ at four visits over a period of 48 weeks. They found that Xeomin^®^ was capable of reducing salivation in both a dose- and time-dependent manner, lasting up to 16 weeks post-injection, without precipitating any adverse events from reinjection. BoNT-B toxins such as Neurobloc^TM^/Myobloc^TM^ can also mitigate sialorrhea in patients with bulbar-onset ALS. Total doses of 1250–3500 U of Myobloc^TM^ reduced excessive salivation as soon as one week after injection, lasting up to 12–13 weeks across two studies [76,77]. A direct comparison of BoNT-A and BoNT-B in a randomized, double-blind pilot study by Guidubaldi and colleagues demonstrated that 250 U of Dysport^®^ or 2500 U of Neurobloc^TM^ were similarly safe and effective in mitigating sialorrhea associated with ALS and PD [78]. In summary, both BoNT-A and -B toxins are similarly well tolerated and effective in the treatment of sialorrhea. Adverse events from either formulation are limited to symptoms including change in saliva viscosity, minor dysphagia, chewing difficulties, and dry mouth [64].

### 4.3. Management of Achalasia

The lower esophageal sphincter (LES) consists of a ring of smooth muscle under sympathetic and parasympathetic regulation to control its relaxation and contraction, respectively [79]. Achalasia is a rare neurodegenerative disorder characterized by increased LES tone, failure of LES relaxation, and impaired esophageal peristalsis [80,81]. Primary achalasia is characterized by the degeneration of the myenteric plexus and disruption of autonomic supply to control sphincter tone [80,81]. Secondary achalasia is caused by esophageal malignancy, infection (Chagas disease), and iatrogenic gastrointestinal surgical complications [82,83]. Complications of achalasia include regurgitation, reflux, and malnutrition [84]. Psychosocially, achalasia can force changes in eating habits such as slower eating and avoidance of social situations involving meals [84]. 

BoNT can be injected with endoscopic guidance at the LES to limit persistent contractions, such as in conditions like achalasia [85]. Currently, formulations of BoNT are reserved for off-label use to treat achalasia, as none are FDA-approved [86].

In a placebo-controlled trial, Pasricha and colleagues were the first to demonstrate that 80 U of Botox^®^ injected endoscopically at the LES decreased mean LES pressure by 33% and increased LES width by 204% after one week compared to saline injection, as assessed by esophageal manometry [87]. This effect lasted for up to six months in 14/21 patients with achalasia [87]. These findings were supported by future studies demonstrating that 80–100 U of Botox^®^, 250 U of Dysport^®^, and 100 U of Xeomin^®^ can treat achalasia symptoms and keep patients in remission for 6–12 months [88,89,90,91]. Studies have also compared the effectiveness of BoNT against pneumatic balloon dilation for the treatment of achalasia. While both treatments are effective, BoNT injections have a shorter lasting effect than pneumatic balloon dilation [92,93,94]. However, there are minimal reported adverse events associated with BoNT injection at the LES compared to pneumatic balloon dilation, which can lead to esophageal perforation in 3% of procedures [87,88,89,90,91,93,94]. In addition, combined therapy consisting of balloon dilation with 100 U of a Chinese BoNT formulation, lanbotulinumtoxinA (Hengli^®^, Lanzhou Biological Products), was shown to be more effective than BoNT or balloon dilation alone for the treatment of esophageal achalasia [95]. Combined therapy resulted in significantly lower LES pressure and improved symptom scores two years after intervention compared to either monotherapy alone, which were comparable in efficacy but were not significantly different prior to intervention at the two-year follow-up [95].

### 4.4. Management of Anal Fissures

Anal fissures are tears within the epithelial lining of the anal canal distal to the dentate line. Anal fissures can be acute or chronic if fissures persist for longer than six weeks [96,97]. The etiology of the development of anal fissures is unclear but is believed to be due to multiple factors, including anal trauma, underlying infection, or disease [97]. Anal fissures may extend into the internal anal sphincter, exposing the smooth muscle and triggering spasms [97]. Indeed, anal fissures are typically associated with increased anal sphincter tone pressure, which can lead to ischemia, poor healing, and longstanding nocturnal and defecatory pain with or without bleeding [96,97]. 

BoNT aims to relieve pain and promote the healing of anal fissures [98]. This is achieved by relieving spasms of the internal anal sphincter, which surrounds the anal canal and is under autonomic control to regulate the passage and continence of feces [98]. Currently, formulations of BoNT are reserved for off-label use to treat anal fissures, as none are FDA-approved [99]. Current management guidelines for chronic anal fissures recommend topical nitrates or calcium channel blockers as first-line therapy; BoNT or internal anal sphincterotomy is reserved for patients failing pharmacologic therapy [100]. 

BoNT was first investigated as a treatment for chronic anal fissures in 1994. Gui and colleagues found that a total of 15 U of Botox^®^ injected intramuscularly into the internal anal sphincter of patients with chronic anal fissures resulted in weakened tone of the internal sphincter, as assessed by anorectal manometry, and fissure healing in 70% of patients at two months of follow-up inspection [101]. Higher doses of Botox^®^ (25–50 U) have a stronger, dose-dependent healing effect on chronic anal fissures (67–96%) and reduced post-defecatory pain [102,103,104]. Changes in resting anal sphincter pressures are typically observed within the first two months; however, maximal voluntary squeeze pressures are unchanged compared to control groups. Botox^®^ is more effective than nitrate therapy for the treatment of chronic anal fissures, demonstrated by reduced resting anal pressures and improved fissure healing in 96% compared to 60% of patients [105]. Dysport^®^ produces comparable effects to Botox^®^ at slightly higher doses, ranging from 60–150 U [106,107]. Commonly reported adverse events of BoNT injection at the internal anal sphincter are limited to mild flatus incontinence, making it a desirable alternative to nitrites, which can cause headache and topical anal burning. Notably, BoNT produces a comparable, albeit temporary, effect to surgical lateral internal sphincterotomy; a procedure that is successful in 90% of patients but has a greater risk of permanent fecal incontinence [107,108].

### 4.5. Management of Neurogenic Detrusor Overactivity

The detrusor muscle is smooth muscle within the bladder wall that controls the micturition and storage of urine by contracting and relaxing, respectively [109,110]. Neurogenic lower urinary tract dysfunction (NLUTD) comprises a series of dysfunctions of the bladder or urethral sphincters following neurological diseases or injury. Common causes of NLUTD include stroke, spinal cord injury (SCI), central nervous system tumors, Parkinson’s Disease, and demyelinating diseases such as multiple sclerosis or transverse myelitis [111]. A primary manifestation of NLUTD is neurogenic detrusor overactivity (NDO). NDO is characterized by dysfunctional autonomic control over the detrusor smooth muscle due to impaired signaling between the CNS and the bladder [112,113]. NDO results in a decreased quality of life from urinary incontinence, pelvic pain, and increased urinary tract infections [114]. Chronic increased bladder pressures can lead to urinary tract damage, vesicoureteral reflux, and hydronephrosis [114]. Oral antimuscarinics are the most widely used treatment for NDO; however, their effectiveness may be limited by lack of adherence and increased adverse events with higher doses [114,115]. 

Intravesical BoNT can mitigate neurogenic detrusor overactivity (NDO) by blocking presynaptic ACh release at the neuromuscular junction of the detrusor [110,113,116]. Currently, only Botox^®^ is FDA-approved for the treatment of NDO [15]. European Union countries can approve the use of Dysport^®^ after promising results from a 2022 clinical trial [21].

Early reports of the application of Botox^®^ as a treatment for NDO in patients emerged in 2000 [117]. It was reported that 200–300 U of intravesical Botox^®^ improves urinary incontinence quality of life, significantly increases cystometric bladder capacity, and decreases maximum voiding detrusor pressure in patients with SCI or multiple sclerosis from six weeks after the first injection up until nine months of follow-up [117,118,119]. Recent studies have investigated the efficacy of other formulations of BoNT-A to treat NDO in cohorts made up of patients with neurological disorders such as SCI, multiple sclerosis, and stroke [120,121]. Dysport^®^ was shown to produce similar effects to Botox^®^ at doses ranging from 500 to 800 U [21,120]. Xeomin^®^ has not been widely studied in the treatment of NDO; however, a recent study reported 200–300 U intravesical Xeomin^®^ injections in participants with NDO-reduced incontinence episodes and increased intermittent catheterization volumes and time between catheterizations [121]. Currently, only Botox^®^ is recommended by the American Urological Association specifically for the treatment of NLUTD in patients with SCI or multiple sclerosis refractory to oral anticholinergics [122]. While BoNT is beneficial in the management of urinary incontinence in people with NLUTD, its suppression of the detrusor can result in increased urinary retention, increased cystometric capacities, and post-void residuals [115,123]. In addition, multiple BoNT injections can result in irritation of the detrusor wall and have been shown to provoke AD in people with SCI [124], although some studies reported no injection-related adverse events [117,121]. Other reported adverse events include hematuria, pyrexia, dysuria, and urinary tract infections [119,125]. The use of Botox^®^ for treating NDO is well-established, however, practitioners must be cognizant of correct dosing and timing of administration. In addition, further investigations may be required for alternative formulations of BoNT prior to their use in practice.

### 4.6. Management of Autonomic Dysreflexia

AD is a life-threatening complication commonly observed in individuals with SCI at or above the sixth thoracic spinal cord level. In AD, a noxious stimulus below the level of injury can trigger a disproportionate sympathetic response, defined as an elevation in systolic blood pressure greater than 20 mmHg from baseline [126,127,128]. Chronic AD is a risk factor for severe complications including ischemic and hemorrhagic stroke, seizures, myocardial infarction, and death [129]. LUT triggers, such as bladder distension, NDO, urinary tract infection, and urological procedures, are common provoking factors for AD [130,131]. The use of BoNT to mitigate the occurrence of AD is a relatively new area of research with promising implications. 

Intravesical injections of BoNT are hypothesized to limit bladder-related AD by preventing NDO, a common trigger for the reflexive sympathetic spinal response that distinguishes AD [132,133]. 

Currently, only off-label use of Botox^®^ has been evaluated in the treatment of AD [113,116,133,134,135]. 

**Figure 3:**
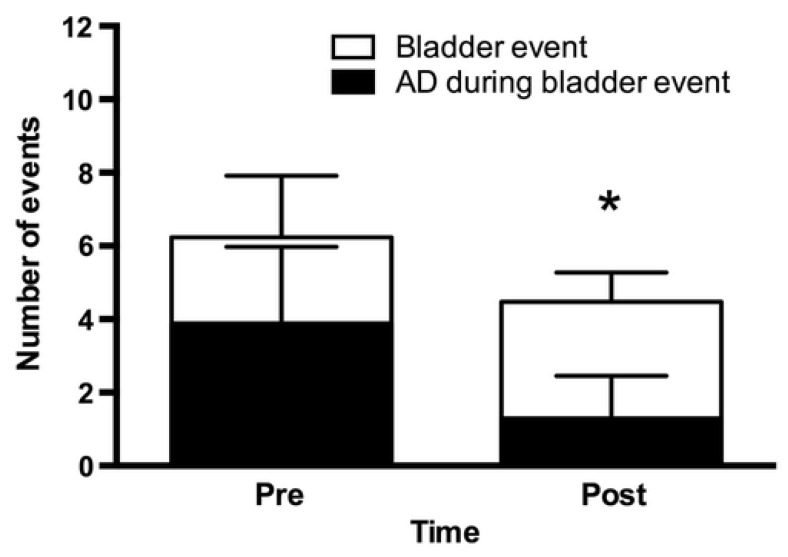
Reduction in bladder-related autonomic dysreflexia events prior to and one month after botulinum toxin injection. Reproduced with permission from Renée J. Fougere et al., 2016, [116], Journal of Neurotrauma; published by Mary Ann Liebert, Inc. The incidence of autonomic dysreflexia (AD) during 24 h blood pressure monitoring prior (Pre) to and one month after (Post) the injection of botulinum toxin. The open bars represent the number of bladder events, and the black bars represent bladder-related AD events. Data are presented as mean ± SD. * *p* < 0.001 for both the number of bladder events and bladder-related AD events.

Urodynamic studies (UDS) are the gold standard for assessing LUT function [136]. UDS involves filling the bladder via catheter while LUT functional parameters are recorded. Catheterization and bladder distension during the procedure can potentially provoke AD [130]. Intravesical injections of 200 U of Botox^®^ diminished bladder-related AD events during UDS [113,116]. This was reported by reduced systolic blood pressure elevations and reduced incidence of self-reported AD symptoms, both during filling and at cystometric capacity, one month after injection [113,133,134,135]. Furthermore, Botox^®^ minimizes the reflexive vagal responses associated with bladder filling during UDS, as evidenced by changes in heart rate variability (HRV) [133]. Parasympathetic HRV parameters were significantly diminished during UDS after BoNT injection, which is associated with the severity of AD [133]. Intravesical Botox^®^ BoNT injections also improved daily bladder-related AD events outside of UDS during 24 h blood pressure monitoring [113,116]. A reduction in the number of AD events and severity was observed over 24 h at the one-month follow-up after BoNT injection (Figure 3) [113,116]. In addition, participants reported improved urinary incontinence-related quality of life after BoNT injection [113,116]. Other formulations of BoNT have yet to be tested for the management of AD. The use of BoNT to treat AD is still in its infancy, yet a number of published clinical trials highlight the potential dual benefit of intravesical BoNT to ameliorate the occurrence of NDO and AD in the SCI population.

### 4.7. Management of Atrial Fibrillation

The rate and rhythm of the heart is modulated by the autonomic nervous system, which can manifest as electrical abnormalities, termed arrhythmias [137]. Atrial fibrillation (AF) is the most common arrhythmia [137,138]. AF can originate and terminate spontaneously (paroxysmal AF) or be persistent and even long-standing when sustained for over 12 months [137]. The incidence of AF after cardiac surgery is estimated to range from 10 to 50% [139]. The complications of postoperative AF are numerous, including hemodynamic instability, cardiac failure, stroke, and kidney injury [140,141]. The current standard treatment for postoperative AF is beta-blockers, which have varying efficacy and may additionally precipitate hemodynamic stability [139,142,143]. 

Recently, off-label injection of BoNT into the epicardial fat pad has received interest for being able to suppress AF after cardiac surgery by modulating the autonomic ganglia contained within [139,143].

In a clinical trial by Romanov and colleagues, a total of 200 U of Xeomin^®^ versus saline was injected across the four cardiac fat pads of patients with a history of paroxysmal AF undergoing coronary artery bypass graft surgery (CABG) [139]. These patients were followed for three years, with the cumulative incidence of any atrial tachyarrhythmia significantly decreased in the BoNT group versus the placebo group [139]. A separate trial by Waldron and colleagues opted to use 250 U of Botox^®^ in a cohort of patients without a prior history of long-standing AF undergoing CABG or valve surgery [143]. They observed a reduction in postoperative AF, although it was not statistically significant [143]. Overall, the number of adverse events from epicardial fat pad injection of BoNT was low [139,143]. In the trial by Romanov and colleagues, events included hospitalization due to recurrent atrial fibrillation, which occurred in 19 patients from the placebo group compared to only two patients who received BoNT [139]. The incidence of adverse events in the trial by Waldron and colleagues did not differ between the BoNT and placebo groups [143]. The discrepancy in results from these studies could be due to BoNT formulation used or the study population, including the history of AF and the type of surgery received [143].

### 4.8. Management of Complex Regional Pain Syndrome

The etiology of complex regional pain syndrome (CRPS) is poorly understood but hypothesized to involve a disruption in coupling between the sympathetic nervous system and peripheral nociceptive fibers [144,145]. The diagnosis of CRPS requires both self-reported symptoms and signs that are evident during clinical examination, including sensory, motor, and autonomic symptoms [144,146]. Type I CRPS presents with symptoms in the absence of a confirmed nerve injury, whereas Type II CRPS requires a known nerve injury, with symptoms typically distal to the site of injury [147]. Long-standing CRPS can result in a plethora of complications, including impaired cognitive function, constitutional symptoms, impaired hemodynamic regulation, urological and gastrointestinal dysfunction [144]. In addition, many patients with CRPS have widespread endocrine dysfunction, in part due to pain management with chronic glucocorticoid and opioid use [144]. 

BoNT is currently a novel experimental treatment to manage the symptoms of CRPS via blocking of sympathetic cholinergic preganglionic fibers following lumber sympathetic ganglion block (LSB) [145,148,149].

The use of BoNT to manage lower extremity CRPS via LSB was first performed in a 2009 clinical trial [148]. Bupivacaine was combined with 75 U of BoNT-A and compared to bupivacaine alone to manage CRPS-related pain. It was found that the combination of bupivacaine and BoNT-A produced a median duration of analgesia of 71 days compared to 10 days with bupivacaine alone [148]. In a separate trial, the effects of BoNT-A (Nabota, Daewoong, South Korea) monotherapy were studied [145]. It was found that 100 U of BoNT-A LSB significantly decreased reported pain scores, in addition to increasing foot temperature compared to the control group, across the 3-month study period [145]. In addition, the effects of LSB 100 U of Botox^®^ and 5000 U of Myobloc^TM^ were compared to manage CRPS [149]. They found that the median duration of analgesia was 15 days for Botox^®^-treated patients and 69 days for Myobloc^TM^ [149]. So far, these are the only three clinical trials to investigate the use of BoNT via LSB to manage lower extremity CRPS-related pain. Apart from one report of temporary nausea and emesis, there were no adverse effects across the three studies [148]. The results of the trials highlight a discrepancy between the duration of effect of BoNT-A; however, it must be noted that the three studies used different formulations or doses of BoNT-A. Overall, BoNT appears to be a safe and effective therapeutic modality to treat lower extremity CRPS; however, future research should be prioritized.

**Table 2:** Summary of the studies, formulations, dosages, and adverse events of botulinum toxin in the treatment of autonomic dysfunctions. U, units; AH, axillary hyperhidrosis; LES, lower esophageal sphincter; BoNT, botulinum toxin; AD, autonomic dysreflexia; HRV, heart rate variability; SBP, systolic blood pressure; BP, blood pressure; ABPM, ambulatory blood pressure monitoring; LSB, lumbar sympathetic ganglion block.

First Author, Year [References]	Study Design, Number (N)	Formulation: Dose	Outcome	Adverse Events
Axillary hyperhidrosis				
Baumann, 2005 [49]	Pilot Study, N = 23	Myobloc^TM^: 2500 U	Improvement of AH from 2.2 to 8.1 months (mean duration 5.0 months)	Bruising, flu-like symptoms, dry eyes, indigestion
Dressler, 2002 [45]	Comparative cohort study, N = 19	Myobloc^TM^: 2000 U	Improvement of AH of a median duration of 17.1 weeks	Mouth dryness, conjunctival irritation
		Myobloc^TM^: 4000 U	Improvement of AH of a median duration of 16.0 weeks	
Heckmann, 2001 [46]	Multicenter clinical trial, N = 145	Dysport^®^: 100 U	Reduced rate of sweat production ≥ 50% at 26-week follow-up	None reported
		Dysport^®^: 200 U	Reduced rate of sweat production ≥ 50% at 26-week follow-up	
Heckmann, 2005 [47]	Randomized clinical trial, N = 43	Dysport^®^: 100 U	Sweat production returned to 98% of baseline 48 weeks after first dose, 63% 48 weeks after second dose	Temporary stinging, irritation, fatigue
		Dysport^®^: 200 U	Sweat production returned to 92% of baseline 48 weeks after first dose, 66% 48 weeks after second dose	
Lowe, 2007 [53]	Multicenter double-blind study, N = 322	Botox^®^: 50 U	Improvement of AH of a median duration of 205 days	None reported
		Botox^®^: 75 U	Improvement of AH of a median duration of 197 days	
Naumann, 2001 [41]	Multicenter, randomized, placebo controlled clinical trial, N = 307	Botox^®^: 50 U	Improvement of AH, as evidenced by decreased sweat production and improved satisfaction scores by 16 weeks compared to placebo control group	Perceived increase in non-axillary sweating, flu-like symptoms
Naumann, 2003 [42]	Prospective double-blind study, N = 207	Botox^®^: 50 U	Improvement of AH of a mean duration of 30.6 weeks between any 2 consecutive treatments and improvements in satisfaction scores.	Perceived increase in non-axillary sweating, flu-like symptoms
Palmar hyperhidrosis				
Campanati, 2014 [50]	Comparative double-blind clinical trial, N = 25	Botox^®^: 100–150 U	Improvement in symptoms and 80% reduction in sweat production by 4 weeks, no difference between formulations	None reported
		Xeomin^®^: 100–150 U	Improvement in symptoms and 80% reduction in sweat production by 4 weeks, no difference between formulations	
Lowe, 2002 [51]	Placebo-controlled study, N = 19	Botox^®^: 100 U	Decrease in sweat production by day 28	Finger tingling and numbness
Moreau, 2003 [31]	Double-blind randomized study, N = 8	Botox^®^: 69 U	Decrease in sweating (−69.4%) significant by 3 months, for a mean duration of 17 weeks	Decreased pinch strength
		Dysport^®^: 284 U	Decrease in sweating (−56.6%) significant by 1 months, but not 3 months (−48.8%) for a mean duration of 18 weeks	
Rystedt, 2013 [52]	Double-blind randomized study, N = 20	Botox^®^: 25 U	Greatest decrease in mean anhidrotic area by 12 weeks at 25 U dose	None reported
		Xeomin^®^: 25 U	Greatest decrease in mean anhidrotic area by 12 weeks at 25 U dose	
		Dysport^®^: 100 U	Greatest decrease in mean anhidrotic area by 12 weeks at 100 U dose	
		Myobloc^TM^: 50 U	Greatest decrease in mean anhidrotic area by 12 weeks at 50 U dose	
Saadia, 2001 [54]	Single-blind randomized study, N = 24	Botox^®^: 50 U	Decrease in sweating by 6-month follow-up	Decreased pinch strength
		Botox^®^: 100 U	Decrease in sweating by 5-month follow-up	
Schnider, 1997 [48]	Double-blind randomized study, N = 11	Dysport^®^: 120 U	Decrease in sweating by 26% at 8 weeks and 31% at 13 weeks	Minor weakness in hand grip strength
Craniofacial hyperhidrosis				
Cabreus, 2019 [60]	Case study, N = 8	Myobloc^TM^: 2250 U	90% median improvement of dermatology quality of life score in treatment group compared to −18% decline in placebo group	None reported
Eustace, 2018 [61]	Case study, N = 20	Botulinum-A toxin (not specified): 100 U, effective at 5–6-month follow-up	Decrease in sweating in 64% of participants, compared to 30% with an oral anticholinergic	None reported
George, 2014 [56]	Case study, N = 4	Botox^®^: 12–80 U, MD 6–8 months	Decrease in sweating in all four participants, duration of effect 6–8 months	None reported
Sialorrhea				
Bhatia, 1999 [70]	Case study, N = 4	Dysport^®^: 20 U	Decrease in salivation of a duration of effect of 6 weeks in one patient and 3–4 months in other patients	Mild dysphagia, chewing difficulty
Costa, 2008 [76]	Open-label prospective study, N = 16	Myobloc^TM^: 1250 U	Reduction in salivation in 94% of patients lasting by 3 months	Increased difficulty chewing, viscous saliva, respiratory infection, facial paresis, burning of eyes
Guidubaldi, 2011 [78]	Prospective, randomized, double-blind, crossover, pilot study, N = 14	Dysport^®^: 250 U	Mean duration of benefit of 75 days, as determined by saliva weight and subjective reporting scales, non-significant compared to BoNT-B	Change in saliva thickness, no difference between formulations
		Neurobloc^TM^: 2500 U	Mean duration of benefit of 90 days, shorter latency of effect compared to BoNT-A	
Isaacson, 2020 [77]	Randomized, parallel, double-blind clinical trial, N = 187	Myobloc^TM^: 2500–3500 U	Reduction in salivation, onset at 1 week after injection, maintained for 13 weeks	Dry mouth, dysphagia, dental caries
Jost, 2019 [74]	Prospective, randomized, double-blind placebo-controlled trial, N = 180	Xeomin^®^: 75–100 U,	Reduction in salivation, still effective at 16 weeks	Dry mouth, dysphagia
Jost, 2020 [75]	Prospective, randomized, double-blind placebo-controlled trial, N = 173	Xeomin^®^: 75–100 U	Reduction in salivation, effective throughout a 64-week period with reinjections every 16 weeks	Dry mouth, dysphagia, speech disorder, worsening constipation
Lagalla, 2006 [71]	Double-blind, randomized, placebo-controlled trial, N = 32	Botulinum-A toxin (not specified): 100 U	Reduction in salivation, effective at 1-month follow-up	None reported
Mazlan, 2015 [72]	Prospective, double-blind, randomized, controlled trial, N = 17	Dysport^®^: 50 U, 100 U, 200 U	Reduction in salivation at 24-week follow-up with the 200 U-treated group showing the greatest effect	None reported
Restivo, 2018 [73]	Randomized, blinded study, N = 90	Botox^®^: 25 U per gland	Reduction in salivation at 2-week follow-up, dose-dependent effect stronger when 4 glands were injected compared to 3	None reported
		Xeomin^®^: 25 U per gland	Reduction in salivation at 2-week follow-up, dose-dependent effect stronger when 3 glands were injected compared to 2	
Achalasia				
Annese, 2001 [88]	Randomized, comparative study, N = 78	Botox^®^: 100 U	Decrease in LES pressure at 1 month and improvement in symptom score lasting by 6 months, no difference between treatments	None reported
		Dysport^®^: 250 U	Decrease in LES pressure at 1 month and improvement in symptom score lasting by 6 months, no difference between treatments	
Jung, 2014 [93]	Non-randomized comparative cohort study, N = 73	Botox^®^: 100 U	Median duration of symptom-free period was 13 months in BoNT-treated group, compared to 29 months in the balloon-dilation-treated group	None reported
Martínek, 2003 [89]	Non-randomized prospective cohort study, N = 41	Botox^®^: 100–250 U Dysport^®^: 100–250 U	Median duration of symptom-free period was 11.5 after first injection, and 10.5 months after second injection among all BoNT-treated patients. Those receiving both BoNT and balloon dilatation had an increased likelihood of remission at 1 and 2 years compared to BoNT alone	None reported
Muehldorfer, 1999 [90]	Prospective randomized study, N = 24	Xeomin^®^: 80 U	All patients receiving successful BoNT treatment experienced symptom recurrence by 6 months, whereas 40% of the balloon dilatation group experienced symptom recurrence	One case of myotomy to remove esophageal adhesions
Pasricha, 1995 [87]	Double-blind clinical trial, N = 21	Not specified: 80 U	Mean decrease in LES pressure of 33% in treatment group compared to an increase of 12% in placebo group; 14 patients receiving BoNT were still in remission by 6 months	None reported
Pasricha, 1996 [91]	Prospective cohort study, N = 31	Not specified: 80 U	Among 19 initial responders, median duration of symptom relapse was 468 days	None reported
Zhu, 2009 [95]	Randomized study, N = 90	Hengli^®^: 100 U	Improved LES pressure and symptom score in BoNT and balloon dilatation combination therapy compared to monotherapy during 2-year follow-up	None reported
Anal fissure				
Berkel, 2014 [106]	Randomized clinical trial, N = 60	Dysport^®^: 60 U	Complete fissure healing of a median duration of 9 weeks in 67% of patients receiving BoNT, compared to 33% of patients treated with isosorbide dinitrate ointment	Headache, loss of mucus, flatus, and mucus incontinence
Brisinda, 1999 [105]	Randomized-blinded clinical trial, N = 50	Botox^®^: 20 U	Complete fissure healing in 96% of patients receiving BoNT at 2 months compared to 60% of patients receiving nitroglycerin	None reported
Brisinda, 2002 [102]	Randomized double-blind clinical trial, N = 150	Botulinum-A toxin (not specified): 20–80 U	Complete fissure healing in 73% of patients receiving 20 U then 30 U of BoNT, and 87% in patients receiving 30 U then 50 U at 1 month, increasing to 89% and 96%, respectively, by 2 months	Mild incontinence of flatus
Brisinda, 2004 [107]	Randomized controlled clinical trial, N = 50	Botox^®^: 50 U	Complete fissure healing in 92% of patients, decrease in mean resting anal pressure (41.8%) and maximum voluntary squeeze pressure (20.2%) compared to baseline at 2 months	Mild incontinence of flatus
		Dysport^®^: 150 U	Complete fissure healing in 94% of patients, decrease in mean resting anal pressure (60.0%) and maximum voluntary squeeze pressure (71.0%) compared to baseline at 2 months	
Gui, 1994 [101]	Case Study, N = 10	Botox^®^: 15 U	Complete fissure healing in 70% of patients at 2 months	Mild incontinence of flatus
Maria, 1998 [104]	Comparative treatment study, N = 57	Botox^®^: 35–45 U	Improved fissure healing at two months in patients treated with 45 U of BoNT (68%) compared to patients treated with 35 U (43%)	None reported
Neurogenic detrusor overactivity				
Asafu-Adjei, 2019 [121]	Pilot study, N = 17	Xeomin^®^: 200–300 U	Decrease in daily pad use, urinary frequency, incontinence episodes, increase in hours between catheterization and catheterization volume, and improvements in symptom score during follow-ups between 2 to 4 weeks	None reported
Chen, 2014 [119]	Randomized, prospective study, N = 72	Botox^®^: 200–300 U	Improvement in incontinence severity and quality of life at 6- and 12-month follow-ups with no difference between 200 U and 300 U dosage groups	Difficult urination, hematuria, urinary tract infection
Grise, 2010 [120]	Prospective, randomized, double-blind, comparative study, N = 77	Dysport^®^: 500–750 U	56.4% of patients receiving 500 U were continent at day 30, compared to 73.7% of patients receiving 750 U	Hematuria, pyelonephritis, urgency, general fatigue with vertigo, difficulty with catheterization
Herschorn, 2011 [118]	Prospective, double-blind study, N = 57	Botox^®^: 300 U	Decrease in number of incontinence episodes at 6-, 24-, 36-week follow-up	Urinary tract infection
Kennelly, 2022 [21]	Two randomized double-blind phase 3 clinical trial, N = 485	Dysport^®^: 600–800 U	Reduction in weekly neurogenic detrusor overactivity incontinence episodes and an increased total voiding volume at 2-, 6-, and 12-week follow ups	Urinary tract infection hematuria, acute pyelonephritis, autonomic dysreflexia
Schurch, 2000 [117]	Prospective non-randomized study, N = 21	Botulinum-A toxin (not specified): 200–300 U	Restoration of continence in 89% of completed participants, along with increases in mean maximum cystometric capacity, reflex volume, and post-void residual volume, as well as a decrease in mean detrusor voiding pressure at 6-week follow up	None reported
Autonomic dysreflexia				
Dorey, 2021 [133]	Secondary post hoc analysis on prospective clinical trial, N = 55	Botox^®^: 200 U	Amelioration in AD-associated HRV responses during bladder filling after 1-month post-injection	Fatigue, pain, urinary tract infection
Fougere, 2016 [116]	Prospective, pre/post comparison study, N = 17	Botox^®^: 200 U, effective at 1-month follow-up	Reduction in change in SBP during bladder filling and the number of bladder-related AD events over 24 h ABPM 1-month post-injection	Headache, urinary tract infection
Huang, 2022 [134]	Cross-sectional, non-randomized clinical trial, N = 25	Botox^®^: 200 U, effective at 3-month follow-up	Decreased maximum detrusor pressure and change in SBP during bladder filling as well as the number of bladder-related AD events over 24 h ABPM 3 months post-injection	None reported
Jung, 2019 [135]	Case study, N = 1	Botox^®^: 200 U	Stabilization of BP and daily maximum SBP 1-month post-injection and improvements in AD symptoms and bladder spams 6 months post-injection	None reported
Walter, 2020 [113]	Prospective clinical trial	Botox^®^: 200 U, effective at 1-month follow-up	Reduction in AD severity in 82% of participants during bladder filling and 74% during 24 h ABPM, increase in cystometric capacity and maximum detrusor pressure at cystometric capacity 1-month post injection	Increased fatigue, headache, pain
Atrial Fibrillation				
Romanov, 2018 [139]	Randomized, double-blind placebo-controlled trial, N = 34	Xeomin^®^: 200 U	Reduced cumulative incidence of atrial tachyarrhythmia over 36 months	Hospitalization due to recurrent atrial fibrillation
Waldron, 2019 [143]	Randomized, double-blind placebo-controlled trial, N = 130	Botox^®^: 250 U	Lower but non-significant incidence of postoperative atrial fibrillation	No difference compared to placebo
Complex Regional Pain Syndrome				
Carroll, 2009 [148]	Randomized, double-blind, controlled, crossover trial, N = 9	Botulinum-A toxin (unspecified): 250 U	Median duration before analgesic failure was 71 days post-LSB in BoNT treatment group compared to 10 days in untreated group	Temporary nausea and emesis in one participant
Lee, 2018 [149]	Retrospective observational trial, N = 18	Botox^®^: 100 U	Median duration before analgesic failure was 15 days	None reported
		Myobloc^TM^: 5000 U	Median duration before analgesic failure was 69 days	
Yoo, 2022 [145]	Randomized, double-blind control trial, N = 48	Botulinum-A toxin (Daewoong, South Korea): 75 U	Improved analgesia over 3-month study period compared to levobupivacaine	None reported

## 5. Discussion

Our search revealed that BoNT is a safe and effective treatment for several autonomic dysfunctions. Despite this, the widespread adoption of BoNT as an approved treatment for autonomic dysfunction is limited (Table 1). Some formulations of BoNT have received FDA or international approval for conditions like Botox^®^ to treat axillary hyperhidrosis or Xeomin^®^ to treat sialorrhea [15,17]. The widespread and clinically approved usage of BoNT for these conditions is backed by the strong level of scientific evidence, exemplified by large-scale randomized control trials with minimal adverse effects [41,42,43,74,75]. Moreover, individuals receiving these treatments have reported improvements in symptoms and quality of life (Table 2). However, this has not yet led to the approval of other widely available formulations of BoNT for these conditions. In contrast, the use of any BoNT formulation to treat achalasia or anal fissures remains limited to off-label use, despite both conditions being longstanding topics of investigation and having clinical trials reporting symptom and quality of life benefits with using BoNT. NDO is another condition with a considerable number of positive outcomes emerging from several studies over many years. Yet, the use of BoNT as a treatment was without formal approval until the results of two large phase 3 clinical trials by Kennelly and colleagues in 2022. Thereafter, Ipsen was granted a positive opinion on the use of Dysport^®^ in the European Union [21]. The lack of approved indications to treat AD, AF, or CRPS is understandable, given the fewer number of published clinical trials that exist. Nevertheless, our review of these studies demonstrates that BoNT could be a promising treatment for these conditions in the future, based on the favorable outcomes associated with its use. 

A striking attribute that is shared across both approved and off-label BoNT formulations is a low or comparable adverse event profile when compared to first-line and alternative treatments for the same conditions. While the incidence and severity of adverse events of BoNT are low, they must still be monitored for patient safety and research reporting. In addition, hypersensitivity to BoNT is a rare yet possible adverse event associated with repeated injections [150]. This was not reported in any of the included studies but is an area of continued research that is focused on developing BoNT formulations that minimize immunogenicity [151,152]. 

Similar to previously published narrative reviews on BoNT for the treatment of autonomic dysfunction, there does not appear to be a consensus on the single dose of BoNT that is most effective in treating most conditions [44]. The duration of the effect of BoNT reported in studies from our search typically ranged from 3 to 6 months, with some studies reporting symptom remission up to a year after injection (Table 2). The timeframe of 3–6 months mirrors the reported duration of effect of BoNT for the treatment of non-autonomic conditions such as in cosmetics and for movement disorders [124,125,126]. The relative consistency in the duration of effect of BoNT observed is interesting, given the variability in dosages that were studied. For example, multiple studies investigating the use of Neurobloc^TM^/Myobloc^TM^ to treat sialorrhea tested doses ranging from 1250 U to 3500 U but observed similar duration of effects between 12 to 13 weeks [76,77]. Similarly, increasing the dose of Botox^®^, Dysport^®^, or Myobloc^TM^ does not result in any statistically significant improvements in mean duration of effect, or the occurrence of adverse events in the treatment of axillary hyperhidrosis [43,45,46,47]. Thus, there appears to be a large dosage range for BoNT that produces similar outcomes. Indeed, it has been previously described that BoNT can reach saturation at higher doses, resulting in insignificant differences in its duration of effect [153]. Furthermore, autonomic conditions that are under-investigated, such as AD, lack experimental diversity in terms of formulations and doses of BoNT studied, as the current literature has focused on testing the effectiveness of 200 U of Botox^®^. The study of other formulations and dosages of BoNT would be useful to determine whether a specific formulation or dosage provides optimal outcomes or not.

Overall, future reviews focused on BoNT treatment of individual autonomic dysfunctions should apply a systematic approach to compare dosages, formulations, and outcomes to determine whether differences truly exist. The optimal, lowest effective dose of BoNT should also be identified. This could help provide insights to help develop future clinical guidelines, particularly for autonomic conditions less studied without current BoNT approval.

## 6. Conclusions

Since its regulatory approval, the adoption of BoNT has garnered prominence and controversy, predominantly from its popularity in cosmetics and later in the management of spasticity related to injury of the CNS. However, its evolution as an effective treatment for several autonomic dysfunctions is equally intriguing. BoNT has widespread applicability in treating several autonomic conditions with comparable or superior efficacy to existing treatments. The lack of adverse events associated with its correct use makes BoNT an appealing treatment option. The remarkable progression of BoNT from a potent neurotoxin to a widely embraced effective therapeutic treatment is reflected in its groundbreaking impact in several areas of medicine, including autonomic dysfunction.

## Figures and Tables

**Table 1:** Summary of the four formulations of botulinum toxin that have received FDA and international approval for application to treat non-cosmetic conditions.

Chemical, Brand Name, and Company	Initial FDA Approval	Dosage	Approved General Indications	Approved Autonomic Indications	Contraindications
OnabotulinumtoxinA, Botox^®^, Allergan Inc. [15]	1989	50 units (U), 100, U, 200 U vials Do not exceed the lesser of 400 U or 7 U/kg in a 3-month interval for adults Do not exceed the lesser of 340 U or 10 U/kg 3-month interval for pediatric patients	Chronic migraine, spasticity, cervical dystonia, blepharospasm, strabismus	Overactive bladder, neurogenic detrusor overactivity, severe axillary hyperhidrosis	Known hypersensitivity to botulinum toxin product or infection at proposed injection site, urinary tract infection, urinary retention for intradetrusor injections, or in patients not willing and able to have clean intermittent catheterization initiated
AbobotulinumtoxinA, Dysport^®^, Ipsen Pharmaceuticals [16]	2009	300 U or 500 U vials	Adult cervical dystonia, spasticity, glabellar lines	Neurogenic detrusor overactivity incontinence (European Union) [21]	Known hypersensitivity to botulinum toxin product, cow’s milk protein, or infection at proposed injection site
IncobotulinumtoxinA, Xeomin^®^, Merz [17]	2010	50 U, 100 U, or 200 U vials	Spasticity, adult cervical dystonia, blepharospasm, hemifacial spasm, glabellar lines	Chronic sialorrhea in adults	Known hypersensitivity to botulinum toxin product or infection at proposed injection site
RimabotulinumtoxinB, Myobloc^TM^ or NeuroBloc^TM^, Solstice Neurosciences [18]	2000	2500 U, 5000 U, 10,000 U vials	Cervical dystonia	Sialorrhea	Known hypersensitivity to botulinum toxin product or infection at proposed injection site

## Data Availability

All data generated and analyzed during this study are included in the published article; further inquiries can be directed to the corresponding author.
